# ﻿Trophobiosis between a new species of *Acropyga* (Hymenoptera, Formicidae) and new *Neochavesia* (Hemiptera, Xenococcidae) from Peru, and establishment of the *Acropygasmithii* species-group

**DOI:** 10.3897/zookeys.1154.97578

**Published:** 2023-03-17

**Authors:** John S. LaPolla, Scott A. Schneider

**Affiliations:** 1 Department of Biological Sciences, Towson University, 8000 York Road, Towson, MD, 21252, USA Towson University Towson United States of America; 2 USDA, Agricultural Research Service, Henry A. Wallace Beltsville Agricultural Research Center, Systematic Entomology Laboratory, 10300 Baltimore Avenue, Beltsville, MD, 20705, USA Agricultural Research Service, Henry A. Wallace Beltsville Agricultural Research Center, Systematic Entomology Laboratory Beltsville United States of America

**Keywords:** Ants, Coccoidea, Coccomorpha, mutualism, root mealybug, taxonomy, symbiosis

## Abstract

We describe a new pair of trophobiotic partners from the ant genus *Acropyga* and the root mealybug genus *Neochavesia*. A recent field study on *Acropyga* ants and associated root mealybugs, conducted in the Peruvian Amazon, led to the discovery of *Acropygamanuense* LaPolla & Schneider, **sp. nov.** and its root mealybug symbiont *Neochavesiapodexuta* Schneider & LaPolla, **sp. nov.** The new root mealybug belongs to the family Xenococcidae, whose members are all obligate associates of *Acropyga* ants. Providing joint descriptions of new mutualist partners in the same article is a novel approach for this system, and it offers benefits to the ongoing study of mutualism and patterns of association among these symbiotic ants and scales. Here, we also begin to revise the species-group composition of *Acropyga* by establishing the *smithii* species-group, and we provide updated information to aid in identifying the new ant species and root mealybug species.

## ﻿Introduction

Efforts to understand the obligate symbiotic relationship between *Acropyga* Roger ants (Hymenoptera: Formicidae) and root mealybugs (Hemiptera: Rhizoecidae and Xenococcidae) hinge on an understanding of exactly which species associate with each other. Questions about fidelity and specificity among partnerships cannot be answered without detailed examination of species associations. However, given the cryptic nature of these mutualists (i.e., they are hypogeic, small, and difficult to identify), definitive associations are often difficult to confirm. Additionally, studies of each respective group are typically segregated by taxonomic discipline, and therefore, information about mutualistic partnerships can be disconnected both physically and temporally in the published record.

Schneider et al. (2022) recently detailed a protocol to confirm direct association between root mealybugs and *Acropyga* ants and reported on several new associations between partnered pairs from the Peruvian Amazon. As part of that field study, we discovered a new species of *Acropyga* associating with a new xenococcid root mealybug. All species in the family Xenococcidae, which is comprised of the Old World genera *Eumyrmococcus* Silvestri, 1926 and *Xenococcus* Silvestri, 1924 and the New World genus *Neochavesia* Williams & Granara de Willink, 1992, are obligatorily associated with *Acropyga*. Past experience has shown that when a new species of *Acropyga* is discovered, it is frequently associated with a new xenococcid as well. This is illustrated by the following (non-exhaustive) list of examples. In the Neotropics, LaPolla (2004) described two new species of *Acropyga* (*A.ayanganna* LaPolla and *A.stenotes* LaPolla) that were each associated with a new species of *Neochavesia* (*N.lapollai* Williams and *N.linealuma* Schneider & LaPolla, respectively). In the Old World, *Acropygakinomurai* Terayama & Hashimoto was discovered associating with a new root mealybug species, *Xenococcuskinomurai* Williams & Terayama (Williams and Terayama 2000); *A.nipponensis* Terayama with *Eumyrmococcusnipponensis* Terayama (Terayama 1986); *A.pallida* (Donisthorpe) with *E.adornocapillus* (Schneider & LaPolla, 2011); and *A.paleartica* Menozzi with *E.corinthiacus* Williams (Williams 1993).

In the above examples, each ant and root mealybug species were described separately from their mutualist partner. Historically, descriptions of new trophobiotic root mealybugs tend to lag behind the descriptions of their mutualist ants, sometimes by many decades. Thus, researchers have had to spend significant effort compiling and reconciling information about species associations scattered across the literature (see Williams 1998; Johnson et al. 2001; LaPolla 2004; Schneider and LaPolla 2011). By combining the methodology outlined in Schneider et al. (2022) with descriptive taxonomy, we aim to present a clearer picture of trophobiotic associations between *Acropyga* and root mealybugs going forward and to keep critical ecological data together in the published record when possible. Here, we describe the trophobiotic association between a new species of *Acropyga* with a new species of *Neochavesia* from Peru.

## ﻿Materials and methods

### ﻿Root mealybug specimens

Specimens were preserved in 95–100% ethanol and stored at –80C prior to preparation and subsequently mounted on glass slides in Canada balsam. Morphological terminology for *Neochavesia* conforms to usage from Williams (2004) and Schneider and LaPolla (2011). Measurements were made on a Zeiss Axio Imager.M2 (Carl Zeiss Microscopy, LLC, White Plains, NY, USA) microscope with the aid of an AxioCam and AxioVision software. Slide-mounted specimens were examined under phase contrast and differential interference contrast (**DIC**) microscopy.

### ﻿Ant specimens

Specimens were preserved in 95–100% ethanol and stored at –80C prior to preparation. Specimens were later point mounted for morphological examination. Ants were measured using a KM33-R micrometer on a Leica MZ16 dissecting microscope to the nearest 0.001 mm. Images were taken with a 10× lens attachment using a Canon EOS 6D Mark II camera with a MP-E 65mm manual focus macro lens on a Macropod Pro 3D and Micro Kit System (Macroscopic Solutions, East Hartford, CT, USA). Images were focus stacked using Zerene Stacker ver. 1.04 software. Morphological terminology used for *Acropyga* description conforms to usage from LaPolla (2004) and LaPolla et al. (2017).

Ant measurements and indices are defined as:

**EL** (Eye Length): In full-face view, maximum anteroposterior length of the compound eye.

**PFL** (Profemur Length): Length of profemur in lateral view.

**HL** (Head Length): In full-face view, length from a line drawn across the posterior margin of the head to a line drawn across the anterior margin of the clypeus.

**HW** (Head Width): In full-face view, maximum width of the head between the lateral margins, excluding the compound eyes.

**ML** (Mesosoma Length): In lateral view, maximum length from the point at which the pronotum meets the cervical shield to the posterior basal angle of the metapleuron.

**PW** (Pronotum Width): In dorsal view, maximum width of the pronotum.

**SL** (Scape Length): In a view perpendicular to the long axis of the scape, maximum length of the scape, excluding the condyle.

**CI** (Cephalic Index): (HW/HL) × 100.

**REL** (Relative Eye Length Index): (EL/HL) × 100.

**SI** (Scape Index): (SL/HW) × 100.

### ﻿Specimen depositories

Type depositories are abbreviated as follows:

**UNMSM**Museo de Historia Natural, Universidad Nacional Mayor de San Marcos, Lima, Peru.

**USNM**Smithsonian National Museum of Natural History, Coccomorpha collection at USDA Agricultural Research Service, Beltsville, Maryland, USA.

**USNM** Smithsonian National Museum of Natural History, Washington, D.C., USA. (ant specimens)

## ﻿Results

### ﻿Establishment of the *Acropygasmithii* species-group

**Diagnosis of *Acropygasmithii* species-group**

New World species; workers with 8 antennal segments and 4 mandibular teeth (one exception occasionally seen in *A.oreithauma* where a much smaller tooth is found between the 3^rd^ and basal teeth); males with penial sclerites elongated with distal tips that bend towards gonopods; anterior portion of the ventral margin of the penial sclerites dentate.

Included species:

*A.fuhrmanni* (Forel, 1914)

*A.manuense* LaPolla & Schneider, sp. nov.

*A.oreithauma* LaPolla, Williams & Fan, 2017

*A.smithii* Forel, 1893

#### Diagnosis and remarks

LaPolla (2004) created nine informal species-groups within *Acropyga* to replace the old subgenera that had previously been recognized within the genus. Those species-groups were largely based on a phylogenetic analysis of male genitalic characters. The most speciose of the New World groups is the *decedens* species-group, with 12 species included by LaPolla (2004) and a thirteenth species (*A.oreithauma* LaPolla, Williams & Fan, 2017) implied to be included by LaPolla et al. (2017), since they considered it a possible sister species to *A.fuhrmanni* (Forel, 1914).

**Figures 1–3.**
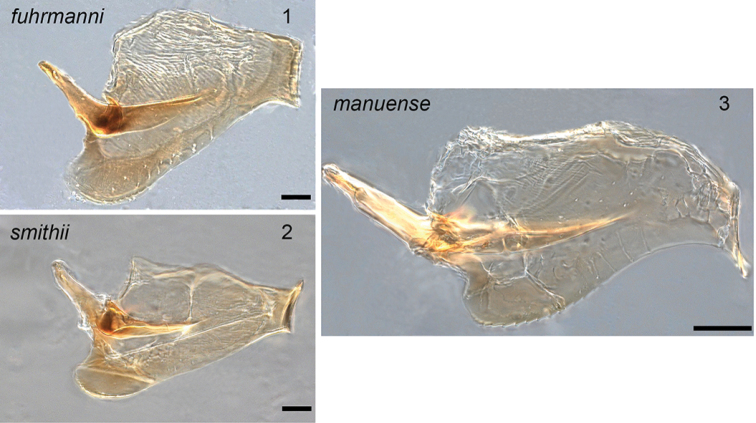
Dissected penial sclerites of various species in ectal view. Scale bar: *fuhrmanni* and *smithii* = 0.03 mm; *manuense* = 0.05 mm.

The prior inclusion of *A.fuhrmanni*, *A.oreithauma* and *A.smithii* Forel, 1893 within the *decedens* species-group is challenged by the discovery of a new species, *Acropygamanuense* LaPolla & Schneider sp. nov., along with additional male specimens of *A.fuhrmanni* and *A.smithii* collected from Peru. Evidence drawn from the morphology of males and workers suggests that these four species form a clade within the New World *Acropyga* that is separate from the *decedens* species-group. The penial sclerites of *A.fuhrmanni*, *A.manuense*, and *A.smithii* share key diagnostic similarities (Figs 1–3). Unfortunately, the male of *A.oreithauma* remains unknown. In all three species the penial sclerites are elongated with distal tips that bend towards the gonopods. These are quite different than the expanded distal tips observed in most of the *decedens* species-group members (the species with such penial sclerites were called the *goeldii* complex by LaPolla (2004) within the *decedens* species-group). The anterior portion of the ventral margin of the penial sclerites also has dentate edges. The workers of *A.fuhrmanni*, *A.manuense*, *A.oreithauma* and *A.smithii* all have 8 antennal segments (occasionally *A.smithii* workers will have as few as 7 segments but this is rarely seen in specimens in collections) and mandibles with 4 mandibular teeth (occasionally a much smaller tooth is found between the 3^rd^ and basal teeth in *A.oreithauma*).

Molecular-based work has suggested that at least *A.fuhrmanni* and *A.smithii* are separated from the *decedens* species-group (Blaimer et al. 2016) and a more recent molecular phylogeny (J. LaPolla, unpub. data) of all described New World *Acropyga* confirms that *A.fuhrmanni*, *A.manuense*, *A.oreithauma* and *A.smithii* comprise a separate clade in alignment with the morphological assessment provided above. Therefore, morphological evidence from both males and workers, combined with molecular results, support the establishment of the *Acropygasmithii* species-group. A revision of the other *Acropyga* species-groups will follow pending publication of molecular phylogenetic results and morphological reassessment.

### ﻿New species descriptions

#### Hymenoptera Linnaeus, 1758

**Formicidae Latreille, 1809**

##### ﻿ Acropyga manuense

Taxon classificationAnimaliaHymenopteraFormicidae

LaPolla & Schneider sp. nov.

4DEDB144-7127-52C6-AF6E-DED4CF043490

https://zoobank.org/1C9F0061-5D0F-49B6-A003-3D71BB9AC4E4

**Figures 4–6.**
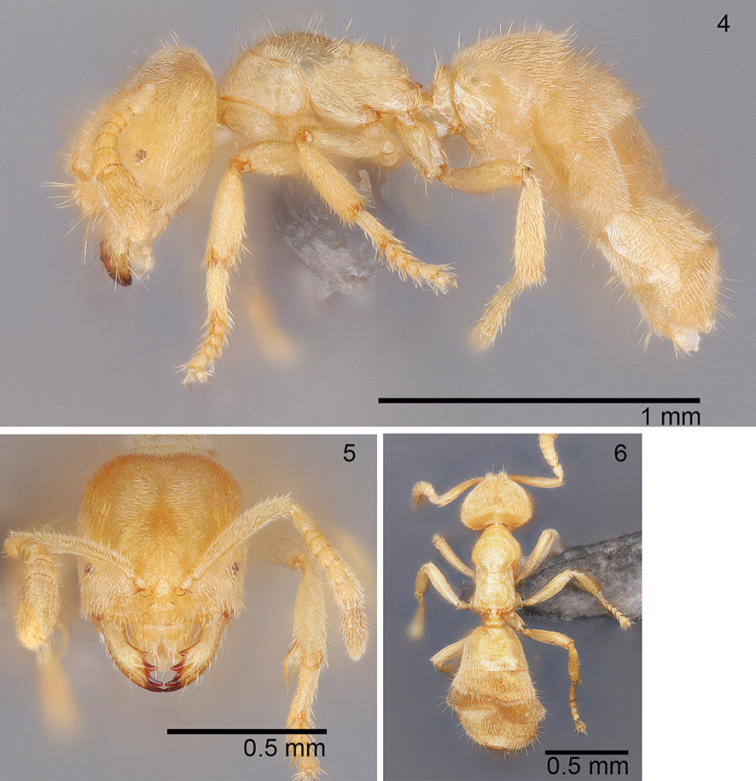
*Acropygamanuense* sp. nov., worker USNMENT01130435 (holotype) **4** lateral view **5** full-face view **6** dorsal view.

Figs 4–6 (worker), 7–8 (queen), 3 and 9–12 (male)

###### Description.

***Worker* (*N = 10*).** Uniformly yellow; covered in a dense layer of pubescence including lateral portions of pronotum and mesopleuron; scattered erect setae across body. Head slightly longer than wide (CI: 90–99); posterior margin slightly concave medially; posterolateral corners rounded with ca. 6 erect setae found along margin; eyes small with uneven pigmentation (REL: 6–11); 8-segmented, incrassate antennae; scapes short of posterior margin by about ¼ to ½ length of pedicel (SI: 71–83); scapes with dense layer of pubescence and scattered erect setae across its length. Clypeus narrow (width in holotype = 0.113 mm) and medially convex. Mandibles with 4 distinct teeth; apical teeth are the longest; teeth 2 and 3 are about equal in size and basal tooth is slightly smaller than teeth 2 and 3; a slight diastema exists between tooth 3 and the basal teeth. In lateral view, mesosoma profile of pronotum steeply rising toward mesonotum (ca. 45° angle if a line is drawn parallel to mesosomal venter). Posterior portion of pronotum and remainder of mesosomal notum with scattered erect setae of varying heights. Highest portion of mesonotum slightly higher than propodeum. Metanotal area distinct with length in holotype = 0.061 mm. Dorsal face of propodeum flat with length in holotype = 0.122 mm; declivitous face steep (ca. 75° angle). Petiole thick and erect with rounded apex; last ¼ of petiole surpasses the most dorsal portion of the propodeal spiracle. Gaster typical of *Acropyga* with thick layer of pubescence and scattered erect setae. Measurements (*N* = 8). HW: 0.432–0.489; HL: 0.46–0.531; EL: 0.031–0.057; ML: 0.467–0.583; PW: 0.277–0.329; PFL: 0.326–0.413; HL+ML: 0.927–1.09.

***Queen* (*N = 4*).** As in worker with modifications expected for caste and with the following differences: CI: 95–100; SI: 78–87; REL: 26–32. Measurements (*N* =3). HW: 0.524–0.59; HL:0.55–0.59; EL: 0.147–0.188; ML: 0.844–1.03; PW: 0.531–0.606; PFL: 0.428–0.454; HL+ML: 1.4–1.62.

***Male* (*N = 4*).** Head yellowish-brown, excluding mandibles and funicular segments which are yellow; remainder of body yellow. Head about as long as wide (CI: 99–108); posterior margin slightly rounded; medially with three prominent ocelli just anterior to posterior margin; posterolateral corners rounded with an indistinct angle and 2–3 erect setae. Compound eyes large, surpassing head margin in full-frontal view (REL: 34–40). 9-segmented incrassate antennae; scapes surpass posterior margin by about length of antennal pedicel (SI: 93–97); scapes covered with dense pubescence and widely scattered short erect setae. Clypeus medially convex (clypeal width in paratype USNMENT01130437 = 0.08 mm long); mandible with three teeth, large apical tooth and two smaller teeth at the basal angle; on some mandibles the two smaller teeth appear to have fused at the basal angle rendering the mandible 2-toothed. Mesosoma covered with dense pubescence and scattered short erect setae on mesoscutum and mesoscutellum. Pronotum small and collar-like with overarching large, rounded mesonotum. Mesoscutellum slightly higher than mesoscutellum in lateral view. Propodeum lower than mesonotum with no separation into dorsal and declivitous faces; propodeum flat (paratype USNMENT01130437 = 0.212 mm long) with a slight slope toward petiole. Petiole thick and erect just surpassing the lowest portion of the propodeum in lateral view. Gaster typical of male *Acropyga* with thick layer of pubescence and scattered erect setae. Gonopods in lateral view tapered to a rounded apex (paratype USNMENT01130437 gonopod length = 0.216 mm long); in dorsal view gonopods medially expanded. Cuspi tubular (paratype USNMENT01130437 = 0.094 mm long); where cuspi meet digiti several peg-like teeth span the surface; several setae extend off of cuspi apex as well; digiti tubular before a distinct right angle bending ventrally where they meet the cuspi; ventral facing portion of digiti taper toward apex becoming needle-like (paratype USNMENT01130437 = 0.108 mm long); apex of digiti visible beyond ventral margin of gonopod in lateral view. Penial sclerites elongate (Fig. 3); ventral margin curves from apex through rounded posterior region; along rounded section of penial sclerites margin dentate; apodeme located medially on anterior end of penial sclerites; apex of penial sclerites bend toward gonopods. Measurements (*N* =3). HW: 0.389–0.399; HL: 0.36–0.4; EL: 0.128–0.155; ML: 0.663–0.692; PW: 0.465–0.494; PFL: 0.412–0.451; HL+ML: 1.04–1.08.

###### Material examined.

***Holotype***: Peru • worker; Madre de Dios, Las Cruces, Manu Paradise Lodge, nest behind lodge, in soil around small rotting branches; 13.055°S, 71.544°W; 31.v.2019; J.S. LaPolla and S.A. Schneider leg.; USNMENT01130435 (UNMSM). ***Paratypes***: Peru • same data as holotype; 9 paratype workers: USNMENT01130438 (USNM), USNMENT01130439 (USNM), USNMENT01130450 (USNM), USNMENT01130451 (USNM), USNMENT01130452 (USNM), USNMENT01130453 (USNM), USNMENT01130454 (USNM), USNMENT01130465 (USNM), USNMENT01130483 (USNM); paratype queens: USNMENT01130436 (USNM), USNMENT01130467 (USNM), USNMENT01130468 (USNM), USNMENT01130480 (USNM); paratype males: USNMENT01130437 (USNM), USNMENT01130466 (USNM), USNMENT01130481 (USNM), USNMENT01130482 (USNM).

###### Etymology.

The epithet is a noun in apposition, referring to its type location near Manu National Park, Peru.

###### Remarks.

The workers of *A.manuense* are similar in overall appearance to both *A.fuhrmanni* and *A.smithii* with all three possessing 8 antennal segments (although *A.smithii* workers can occasionally be found with as few as 7 segments) and 4 mandibular teeth. One of the most obvious ways *A.manuense* workers differ from both *A.fuhrmanni* and *A.smithii* workers is in mesosomal pubescence. In *A.manuense* workers possess thick pubescence that extends down the lateral portions of the pronotum and onto the mesopleuron. In *A.fuhrmanni* and *A.smithii* pubescence is very sparse to lacking on the lateral portions of the pronotum and the mesopleuron.

**Figures 7, 8.**
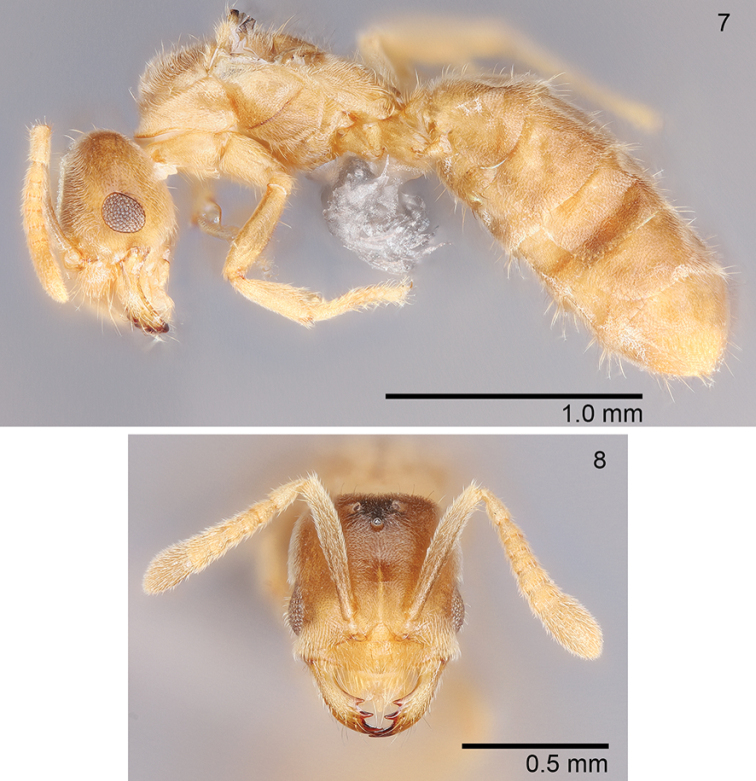
*Acropygamanuense* sp. nov., queen USNMENT01130436 **7** lateral view **8** full-face view.

**Figures 9–12.**
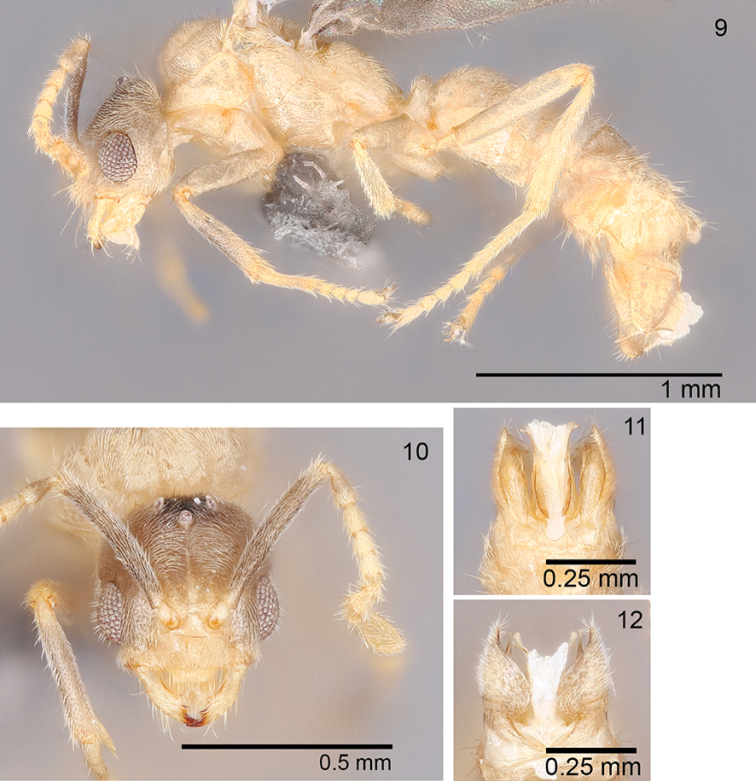
*Acropygamanuense* sp. nov., male USNMENT01130437 **9** lateral view **10** full-face view **11** ventral view of genitalia **12** dorsal view of genitalia.

**Figure 13.**
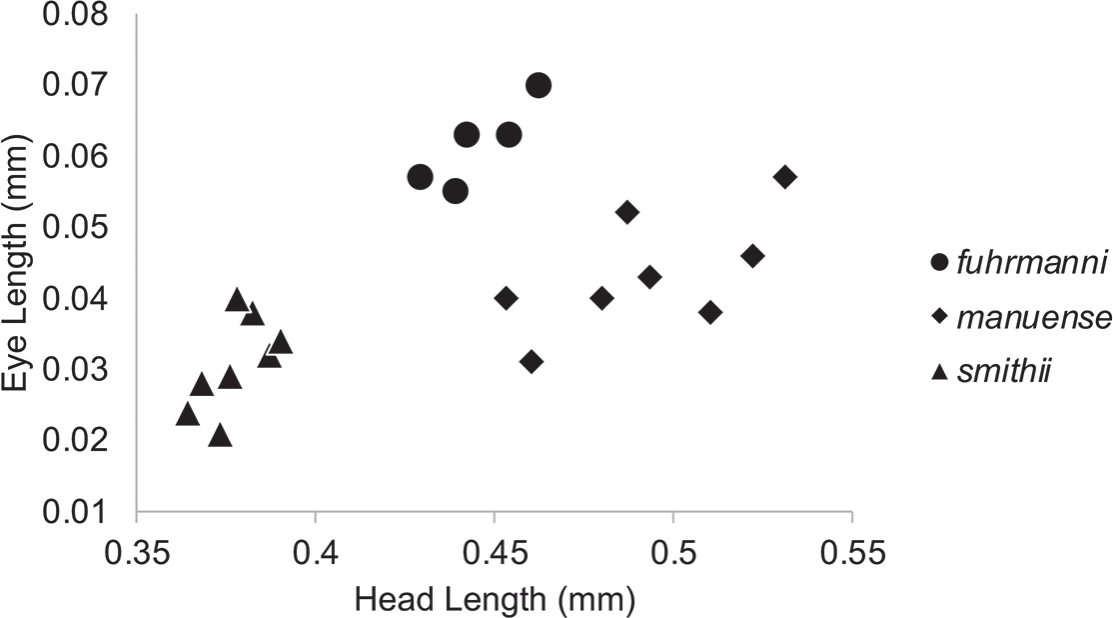
Bivariate plot of eye length vs. head length among measured *A.fuhrmanni*, *A.manuense* and *A.smithii* workers.

Several measurements will also allow for separation of the three species. The smallest of the three species is *A.smithii* with a head width and length less than 0.4 mm, whereas both *A.fuhrmanni* and *A.manuense* have head widths and lengths greater than 0.4 mm. The scape length in *A.smithii* is less than 0.27 mm, while it is greater than 0.3 mm in both *A.fuhrmanni* and *A.manuense*. The small size of *A.smithii* makes it fairly easy to distinguish from both *A.fuhrmanni* and *A.manuense*. Workers of *A.fuhrmanni* and *A.manuense* possesses different eye sizes with a relative eye length index between 12–15 in *A.fuhrmanni* and between 6–11 in *A.manuense* (Fig. 13). The cephalic index is also instructive with *A.fuhrmanni* generally having a value over 100 with *A.manuense* less than 100.

The most striking differences between *A.fuhrmanni*, *A.smithii* and *A.manuense* are found in males. The mandibles of the three species’ males differ in the number and size of teeth, with *A.manuense* possessing three distinct equally sized teeth. Both *A.fuhrmanni* and *A.smithii* only have two teeth: a prominent apical tooth and a much smaller tooth at the basal angle. There are several genitalic differences between the three species such as each possessing uniquely shaped penial sclerites (Figs 1–3). The digitus is very distinctive within *A.manuense*, being ventrally directed, elongated and coming to a prominent point (Figs 9, 11). The elongated, pointed structure of the digitus is unique among all *Acropyga* species.

In the key to New World workers provided by LaPolla (2004), specimens of *A.manuense* will key to the lug for *A.fuhrmanni*. That lug would become a new couplet as follows:

**Table:** 

1	Lateral portions of pronotum and mesopleuron with spare to no pubescence; ocular index between 12–15; cephalic index above 100	***Acropygafuhrmanni* (Forel)**
–	Lateral portions of pronotum and mesopleuron with thick layer of pubescence; ocular index between 6–11; cephalic index less than 100	***Acropygamanuense* LaPolla & Schneider, sp. nov.**

#### Hemiptera Linnaeus, 1758

**Xenococcidae Tang, 1992**

##### ﻿ Neochavesia podexuta

Taxon classificationAnimaliaHemipteraXenococcidae

Schneider & LaPolla sp. nov.

958B1623-C86E-5320-AB6D-A41334FBEE1A

https://zoobank.org/EA866C98-2812-44DC-8B50-0A59D80755B5

Fig. 14

###### Description.

***Adult female* (*N = 7*).** In life, body bright white to cream colored and free of wax. Mounted on microscope slide, body elongate-pyriform, 1.35–1.58 mm long, 0.55–0.73 mm wide at widest point; head and thorax dilated with widest point at metathorax and abdominal segment I. Abdomen constricted after segment III; segments IV–VII gently tapering in width posteriorly with another constriction between segments VII and VIII. Dorsal posterior half of abdominal segment VIII sclerotized; anterior half membranous and free of setae, forming a distinctive bald patch; width of segment 145 μm wide. Anal lobes well developed and separated from abdominal segment VIII on venter and margins of dorsum by an intersegmental line. Anal lobes diverging with a roughly U-shaped notch between them, each rounded at posterior end. Dorsum of each anal lobe with numerous long flagellate setae, longest about 135 μm, situated at posterior end, with those at anterior end about 35 μm; ventral surface with similar setae 32–90 μm long. Anal ring roughly triangular, without cells or setae, 65 μm wide; anterior end lying along the intersegmental division that separates the anal lobes from abdominal segment VIII, posterior edge removed from the apical notch between lobes by about 1X length of anal ring. Long antennae widely spaced on dorsal head margin; basal segment set into a notch on the head and articulating; each with four segments, 652–663 μm in overall length; average lengths of segments (base to apex) 56 μm, 297 μm, 95 μm, 215 μm; the apical segment appearing partially divided, indicating an obsolete fifth segment; few flagellate setae on basal antennal segment, numerous such setae on all other segments, 30–55 μm long. Legs well developed; average length of metatrochanter + femur 212 μm long; metatibia + tarsus 150 μm long; tarsus swollen basally and abruptly tapering; with metaclaw 70–80 μm long, longer than tarsus. Ratio of length of metatibia to tarsus, 1.60; leg segments with multiple stout flagellate setae. Labium 3-segmented, 147 μm long, longer than clypeolabral shield, 70 μm wide; basal segment with three pairs of setae; eight pairs of setae on terminal segment. One round circulus present, situated towards center of abdominal segment II, 16 μm in diameter, conical and projecting from derm, cup-shaped internally. Spiracles normal, 35 μm in diameter at widest point.

**Figure 14.**
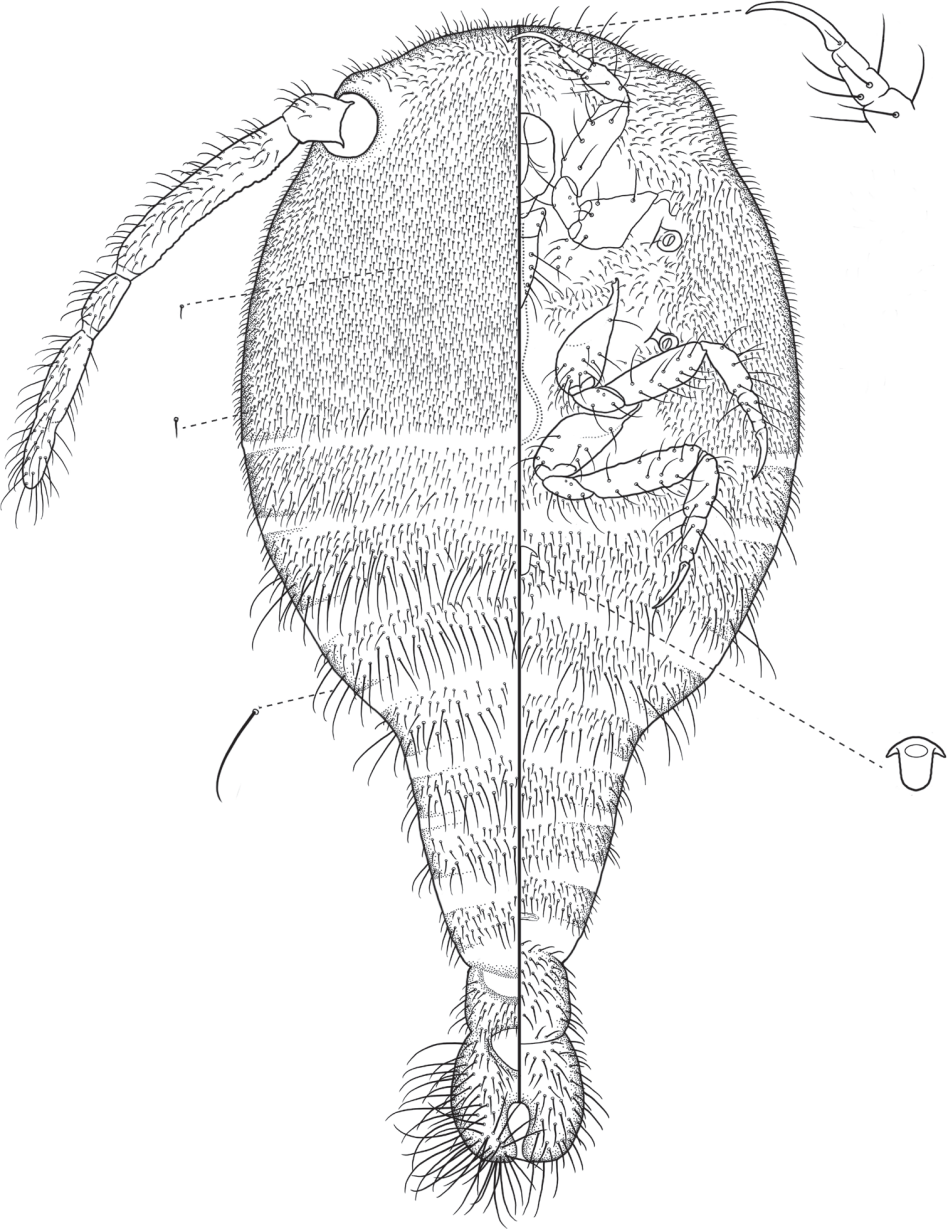
*Neochavesiapodexuta* sp. nov. Adult female, full body view, illustrated from the holotype and paratypes. Illustration by Taina Litwak (USDA, ARS, Systematic Entomology Laboratory) with edits by SAS.

Dorsal surface of head and thorax crowded with slender flagellate setae, about 17 μm long, few approaching 45 μm at posterior end of metathorax, most with small setal collars, longer setae with slightly wider collars; abdominal segments with similar setae but less densely crowded after segment I; on each segment, setae at anterior end shorter and finer than posterior setae, ranging from 19–75 μm on segment I, upwards of 95 μm on segments II–VI, shorter setae 25–40 μm on VII–VIII, longest setae on anal lobes, from 32–135 μm. Venter with similar setation; less densely crowded on head and with greater variation in setal lengths, 15–40 μm; thoracic margin to submargin similar to dorsum; thoracic submedian similar to ventral head; abdominal segments I–IV similar to dorsum, segments V–VIII with shorter setae, anal lobes with numerous long setae but generally shorter than those found on dorsum. Pores and ducts absent.

###### Material examined.

***Holotype***: Peru • 1 adult female; Madre de Dios, Las Cruces, Manu Paradise Lodge, from *Acropygamanuense* nest behind lodge, in soil around small rotting branches; 13.055°S, 71.544°W; 31.v.2019; J.S. LaPolla and S.A. Schneider leg.; UNMSM (nest ID PER01-02; prep S0401E). ***Paratypes***: Peru • 6 adult females; same data as holotype; USNM (nest ID PER01-01; preps S0400A,B,C,D,E,F) • 1 immature female; same data as holotype; USNM (nest ID PER01-02; prep S0401A).

###### Informal synonyms.

This species was previously referred to as “*Neochavesia* undescribed” in Schneider et al. (2022).

###### Etymology.

The epithet is a noun in apposition, referring to the distinctive bald patch located just anterior to the anal opening. The Latin ‘*podex*’, meaning fundament/anus, was combined with ‘*exutus*’, meaning bared or stripped.

###### Remarks.

*Neochavesiapodexuta* bears a distinctive bald patch on the dorsal anterior surface of abdominal segment VIII. On slide-mounted specimens, it often appears as though this segment, including the anal lobes, has become detached from the rest of the body although it is still intact. In life, species of *Neochavesia* hold their abdomen in a curled position over their dorsum, resembling the tail of a scorpion; this bald membranous patch is located where the cuticle would curve inward. Adult females of *Neochavesiapodexuta* are similar to *N.cephalonodus*, *N.eversi*, *N.iwokramae*, and *N.lapollai* in possessing antennae that articulate at the basal segment with a sclerotized prominence (forming a socket) on the head. They also lack a pair of setae on the middle labial segment, a characteristic shared among these species as well. The new species is most akin to *N.eversi*; the former can be distinguished from the latter by the bald patch on VIII described above and by their longer antennae. In *N.podexuta*, the body is about 2.0–2.5 times longer than the antennae with the second segment almost 300 μm long; whereas in *N.eversi*, the body is roughly 3.5 times the length of the antennae with the second segment only about 150 μm long.

Direct association between *A.manuense* and *N.podexuta* was confirmed by collecting ants and root mealybugs into a nest-box and observing interactions (Schneider et al. 2022). Ants gathered root mealybugs into protective clusters within the nest-box and were observed actively tending them. These observations were made over a 48-hour period in field-based laboratory conditions.

### ﻿Key to adult females of *Neochavesia* Williams & Granara de Willink, 1992

Adapted from Schneider and LaPolla (2011) and Williams (2004).

**Table:** 

1	Anal lobes fused with abdominal segment VIII, without intersegmental line; antennae situated toward ventral head margin	**2**
–	Anal lobes separated from abdominal segment VIII by intersegmental line; antennae situated on lateral margin or dorsally on head	**4**
2	Trilocular pores present on head and thorax	***Neochavesiacaldasiae* (Balachowsky)**
–	Trilocular pores absent	**3**
3	Claws over 1/2 length of tarsi; one circulus present on third abdominal segment; posterior abdominal segments with rows of rigid thorn-like setae	***Neochavesialinealuma* Schneider & LaPolla**
–	Claws between 1/4 and 1/3 length of tarsi; two circuli present on abdominal segments II and III; all setae on abdominal segments flagellate	***Neochavesiaweberi* (Beardsley)**
4	Antennae normally five segmented	**5**
–	Antennae normally four segmented	**7**
5	Second antennal segment 203–293 μm long; anal ring transversely elliptical and situated between anal lobes and abdominal segment VIII; many long stout setae on head margin	***Neochavesialapollai* Williams**
–	Second antennal segment 81–185 μm long; anal ring round to triangular and situated near proximal base or in middle of anal lobes between anterior edge and base of U-shaped notch between anal lobes; head without long stout setae	**6**
6	Second antennal segment 81–95 μm long, antennae articulating with prominences on head; anal ring situated in middle of anal lobes between anterior edge and base of U-shaped notch between anal lobes	***Neochavesiaiwokramae* Williams**
–	Second antennal segment 168–185 μm long, antennae not articulating with prominence on head; anal ring situated near proximal base of anal lobes	***Neochavesiatrinidadensis* (Beardsley)**
7	Claws shorter than tarsi; second antennal segment 365–415 μm long; prominences enlarged giving head a swollen appearance	***Neochavesiacephalonodus* Schneider & LaPolla**
–	Claws longer than tarsi; second antennal segment not exceeding 300 μm in length, prominences on head relatively small	**8**
8	Body roughly 2.0–2.5 times longer than antennae, with second antennal segment almost 300 μm in length; dorsal anterior surface of abdominal segment VIII membranous and free of setae, appearing bald	***Neochavesiapodexuta* Schneider & LaPolla, sp. nov.**
–	Body roughly 3.5 times longer than antennae, with second antennal segment about 150 μm in length; dorsal anterior surface of abdominal segment VIII sclerotized and bearing setae	***Neochavesiaeversi* (Beardsley)**

## Supplementary Material

XML Treatment for Acropyga manuense

XML Treatment for Neochavesia podexuta
